# The influence of emotional labor and emotional intelligence on cesarean section decision-making among midwives and obstetricians in Kosovo: A cross-sectional study using conjoint analysis

**DOI:** 10.18332/ejm/197168

**Published:** 2025-01-17

**Authors:** Besarta Taganoviq, Pam Smith, Mateja Lorber, Ilir Hoxha

**Affiliations:** 1 University of Maribor, Maribor, Slovenia; 2 Heimerer College, Prishtina, Kosovo; 3 University of Edinburgh, Edinburgh, United Kingdom; 4 The Dartmouth Institute for Health Policy and Clinical Practice, Lebanon, United States; 5 Evidence Synthesis Group, Prishtina, Kosovo

**Keywords:** cesarean section, emotional labor, emotional intelligence, midwives, obstetricians, decision-making

## Abstract

**INTRODUCTION:**

Cesarean section rates continue to increase worldwide. In 2021, one in every five deliveries was delivered by cesarean section. This is particularly alarming in resource-limited countries such as Kosovo, where the rates continue to increase and vary considerably between hospitals. Understanding the underlying factors that drive the increase and variation of cesarean section rates may help to change these trends. This study investigates how emotional intelligence and emotional labor impact cesarean section decision-making among midwives and obstetricians in Kosovo, along with clinical factors.

**METHODS:**

We employed a conjoint analysis using a cross-sectional study design to assess preferences that drive decisions for cesarean section. We used the Dutch questionnaire on Emotional Labor, the Assessing Emotions Scale, and the Quality of Decision-making questionnaire, and designed a conjoint questionnaire with 28 hypothetical scenarios. We invited all midwives and obstetricians employed at the Gynecology and Obstetrics Clinic of the University Clinical Centre of Kosovo to participate in the study. The data were collected from January to the end of March 2023. Stata 18 BE was used for statistical computing and data visualization.

**RESULTS:**

A gestational age of 42 weeks decreased CS likelihood among midwives (OR=0.75; 95% CI: 0.62–0.90, p=0.002). Previous cesarean sections (OR=1.42; 95% CI: 1.11–1.81, p=0.005) and hypertension (OR=1.23; 95% CI: 1.01–1.51, p=0.042) raised CS odds for midwives. A pelvic size of 8 cm significantly increased CS likelihood for midwives (OR=1.70; 95% CI: 1.37–2.09, p<0.001), while a size of 11 cm was protective for both groups (midwives: OR=0.73; 95% CI: 0.57–0.93, p=0.010; obstetricians: OR=0.70; 95% CI: 0.52–0.94, p=0.019). Maternal age of 40 years was significant only for obstetricians (OR=1.43; 95% CI: 1.00–2.06, p=0.052), and university education was significant for midwives (OR=1.19; 95% CI: 1.03–1.37, p=0.020). Non-clinical factors and emotional measures showed no significant or consistent trends in either group.

**CONCLUSIONS:**

Various clinical and non-clinical factors shape the decision to recommend a cesarean section, with obstetricians and midwives prioritizing these factors differently. These findings underscore the importance of implementing evidence-based practices to enhance maternal and newborn health outcomes in Kosovo and similar settings, while optimizing cesarean decision-making.

## INTRODUCTION

The increasing prevalence of cesarean sections (CSs) continues to be a significant cause for concern in the maternal health domain of service delivery^1^. As of 2021, approximately one in five births worldwide (21%) are delivered by cesarean section (CS)^1^, with a significant proportion deemed unnecessary, an estimated 8.8 million cases in 2018^2^. This trend will likely continue. In 2030, 29% of all births are expected to be delivered with CS^1^. Kosovo, an upper middle-income country in Southeastern Europe, has a relatively high birth rate. In 2021, the proportion of cesarean section (CS) births in public hospitals varied significantly, ranging from 16.2% to 41.1%, and 62.6% to 83.9% in private hospitals^3^.

To improve healthcare quality, particularly in resource-limited settings, issues of underuse and overuse must be addressed^4,5^. Understanding the dynamics underlying the use of procedures like CS should be based on a holistic understanding of the health system, including macro-, meso-, and micro-level factors^6^. Notably, such influences on the likelihood of CS can operate independently of clinical indications for CS^7^. At the macro level, i.e. at the country and health system level, factors such as health insurance status or cultural factors may influence CS decisions^7-9^. For example, Nakamura-Pereira et al.^12^ reported that public or private healthcare funding affected CS rates regardless of Robson group. This underlines the importance of incentive structures for payment methods to steer CS provision towards clinical indications^10^. At the meso level, i.e. at the facility and hospital level, factors, such as for-profit or teaching status hospitals, affecting incentive structure and quality of care control mechanisms can influence the rates^10,11^. At the micro level, i.e. patient, physician, and clinical unit level, factors, such as office hours, have been observed to affect the possibility of CS, likely reflecting the physician convenience effect^13^. Practice patterns that are not based on evidence can also have an effect^7^. For example, using a previous cesarean section as an indication for CS, although the American College of Obstetricians and Gynecologists cautions against using previous cesarean sections as a predictor in non-emergency scenarios^14^. Fetal distress without documented monitoring, labor abnormalities, and a completed partograph have also been observed as a common indication for CS^14^. Furthermore, we can see the correlation between nulliparity and cesarean delivery, which raises concern because it implies that women who have never given birth are increasingly undergoing CS^15^. Other provider and client characteristics and preferences also play a role^7^.

Recent evidence shows that female physicians and midwives who deliver care decrease the rate of CS^16,17^. This may reflect different practice patterns and clinical decision-making approaches. Could that be related to providers’ emotional labor and emotional intelligence? Clinician decision-making is a central moment in CS provision and birthing modes in general^6,18^. As such, this study aims to examine the decision-making process for CS in midwives and obstetricians, considering the quality of decision-making, emotional labor and emotional intelligence as potential factors that may explain the differences in clinical patterns. Emotional Intelligence (EI) has been shown to improve intuitive decision-making^19^. In nursing, research findings increasingly support the importance of EI, linking it with care, empathy, and clinical performance measures^20,21^. According to the literature, emotional labor significantly improved intuitive decision-making^22,23^. As tacit knowledge plays a major role in the interaction between cognitive and affective processes, intuition can be understood as the outcome of personal and environmental awareness. Individuals with an intuitive decision-making style focus more on the larger picture than the specifics^22,23^.

Despite growing research on decision-making in cesarean deliveries, there is a notable gap in the literature regarding the roles of quality of decision-making, emotional intelligence and emotional labor among healthcare providers in CS rates. Hence, this study examines the quality of decision-making, emotional intelligence and emotional labor in midwives and obstetricians and how they affect decision-making concerning CS. The main research question is whether the quality of decision-making, emotional intelligence, and emotional labor influence the decision for CS among midwives and obstetricians. There were two specific study hypotheses. First, we hypothesized that the quality of decision-making, emotional intelligence, and emotional labor scores may be associated with a decreased tendency to recommend CS. Secondly, we hypothesized that clinical factors such as previous CS may increase the probability of a clinician choosing CS as a delivery mode.

## METHODS

### Study design and setting

The study employed a cross-sectional design incorporating conjoint analysis as a central element of the study design. The data were collected in the University Clinical Centre of Kosovo (Clinic of Gynecology and Obstetrics). The center is a public institution and serves as the leading tertiary care clinic in the country. The data were collected from January to March 2023.

### Participants

**Figure 1:**
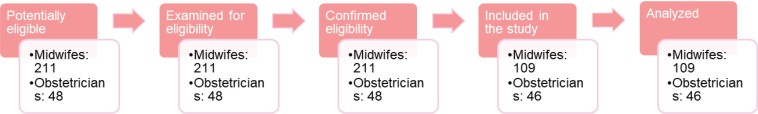
Flow diagram of participants in each stage of the cross-sectional study on emotional labor and emotional intelligence influencing clinical decision-making among midwives and obstetricians in Kosovo, 2023 (N=155)

We included full-time employees, midwives, and obstetricians at the University Clinical Centre of Kosovo with a valid midwife or obstetrician license. We targeted all eligible participants, i.e. all eligible midwives (n=211) and obstetricians (n=48) working at the center, who were evaluated for eligibility (Figure 1).

### Data sources and measurement

We utilized various research instruments to gather the data, consolidating them into a single, well-structured questionnaire. The first section of the instrument was designed to collect demographic and general data about the participants, including variables such as age, gender, as well as information regarding their working experience, such as clinical unit or number of client visits per day. We integrated three additional standardized questionnaires.

We used the Dutch questionnaire on Emotional Labor (D-QEL)^[Bibr CIT0024]24^ to explore how people deal with their emotions at work. It assesses several key areas covering 13 questions: deep acting (3), emotional consonance (2), surface acting (5), and suppression (3). Questions use a Likert scale with the following responses: 1=‘never’, 2=‘sometimes’,3=‘regularly’, 4=‘often’, and 5=‘always’^24^. The questionnaire achieved a Cronbach’s alpha value of 0.836 when piloted in Kosovo, indicating high internal consistency.

To assess the clinician’s level of emotional intelligence, we used the Assessing Emotions Scale, also known as the Emotional Intelligence Scale, the Self-Report Emotional Intelligence Test, or the Schutte Emotional Intelligence Scale^25,26^. The Emotional Intelligence Scale comprises 33 questions, each measured in a Likert scale with the following response options: 1=‘strongly disagree’, 2=‘somewhat disagree’, 3=‘neither agree nor disagree’, 4=‘somewhat agree’, and 5=‘strongly agree’. The Cronbach’s alpha value for the Assessing Emotions Scale was 0.869, indicating excellent internal consistency.

To assess the quality of decision-making, we used the Quality of the Decision-making questionnaire developed by Bujar et al^27-29^. This instrument contains two main parts, with two question sections in each part. The first part measures the organizational level influences; the second part measures the individual level influences. The possible responses are again measured on the Likert scale as follows: 1=‘not at all’, 2=‘sometimes’, 3=‘frequently’, 4=‘often’, and 5=‘always’. The Quality of the Decision-making questionnaire is easy to understand, allowing it to be completed in a short time. In the Kosovo context, the questionnaire achieved a Cronbach’s alpha value of 0.699, indicating an acceptable level of internal consistency. The last section of the questionnaire recorded responses to the conjoint design.

### Conjoint analysis design, attributes and levels

The attributes were chosen based on the research of Danishevski et al.^30^ with adaptations that match the local context (Table 1). We used the orthogonal design to generate our hypothetical scenarios utilizing IBM SPSS Statistics V.22.0 software (IBM Corp., Armonk, NY, USA) orthogonal design facility. In addition to the 26 hypothetical scenarios generated by the respective design, we added two: a scenario aimed to prompt respondents to consider a recommendation for CS and a duplicate scenario of one of the 26 initial ones. The intention was to assess the insistency and reliability of responses, thus validating the integrity of the choice-based analysis. We have tested the design. Using correlation analysis, no evidence of multicollinearity among attributes was found. We presented participants with 28 clinical scenarios, which varied in terms of several key attributes, including fetal weight, weeks of pregnancy, parity and previous CS, pre-existing conditions such as diabetes and hypertension, pelvic size, time of delivery, i.e. office or out of office hours, and the age and education level of the patient (Table 1). The levels of each attribute varied across scenarios. Higher levels, which we used, i.e. 4500 g versus 4200 g in case of fetal weight and other attributes, increase the variability of such attributes, potentially amplifying their impact in the conjoint analysis results^30^. For each scenario, the participants were requested to determine whether they would recommend CS.

**Table 1:** Included attributes and levels of conjoint experiment in a cross-sectional study of emotional labor and emotional intelligence influencing CS decision among midwives and obstetricians conducted in Kosovo, 2023 (N=155)

*Attributes*	*Levels*
Expected birthweight (g)	2500
	3500
	4500
Length of gestation (weeks)	35
	37
	42
Previous pregnancies	Previous CS
	Multipara
	Nullipara
Conditions	None
	Hypertension
	Diabetes
Pelvic outlet size (cm)	8
	10
	11
Time of the day	Working hours (7 a.m. – 2 p.m.)
	Out of office hours (3 p.m. – 7 a.m.)
Maternal age (years)	18
	29
	40
Education level	Primary and secondary education University enrolment or degree

### Translation and testing

Before adopting the questionnaires for our study, we translated them into Albanian. The translation ensured the meaning in Albanian closely matched the original language, and we used back-translation to check how closely the meaning corresponded to the original language and pilot the instruments prior to use^31^. Eighty-five healthcare professionals who met the inclusion criteria participated in the pilot phase conducted from October to November 2022.

### Statistical analysis

First, we conducted a descriptive analysis of the respondents. Multilevel random intercept design and robust variance, using individual caregivers (midwives or obstetricians) as the upper level and clinical attribute levels of individual scenarios as the lower level estimates, were utilized as the primary analysis to investigate the impact of various clinical signs and attributes from the conjoint experiment in the decision-making for the cesarean section. We used several models for analysis. The first model assessed only the clinical attributes from the conjoint experiment. Other models examined different variables related to the health professionals themselves (age, work experience, sector of engagement, quality of decision-making coefficient, emotional labor coefficient, emotional intelligence coefficient) in integrated models with clinical characteristics from the conjoint experiment. We created coefficients for the quality of decision-making, emotional labor and emotional intelligence. The coefficients were average scores of the responses to all questions of the instruments used. We transformed this variable into a categorical variable containing four quartiles for analysis. Odds ratios (ORs) are presented for each characteristic analyzed. Statistical analysis was conducted using Stata/BE 18.0 (StataCorp LLC, 2023). Statistical significance was set at p<0.05.

### Ethical considerations

The study was authorized by the Kosovo University Clinical Centre Service management, which allowed us to collect the quantitative data from the midwives and obstetricians who work in the Gynecology and Obstetrics Clinic (Prot. No. 1745/17 archived on 09.08.2021). We also obtained an ethics approval from the Kosovo Chamber of Nurses, Midwives and Other Health Professionals (Prot. No. 72 archived on 04.02.2022).

## RESULTS

The final sample consisted of 155 participants, including 109 midwives, i.e. 51.7% response rate, and 46 obstetricians, i.e. 95.8% response rate (Figure 1). Midwives’ and obstetricians’ characteristics are presented in Table 2. Among midwives, 56.88% were aged ≤39 years, 35.78% were aged 40–59 years, and 7.34% were aged 60–65 years. Among obstetricians, 17.39% were aged up to ≤39 years, and most, i.e. 71.74%, were aged 40–59 years, and 10.87% were aged 60–65 years. All midwives were female, whereas 58.7% of obstetricians were male and 41.3% were female. Regarding work experience, the majority, i.e. 66.06% of midwives, had ≤10 years of experience, 22.94% had 11–20 years, and 11.01% had >20 years.

**Table 2:** Participant characteristics and scores in a cross-sectional study of emotional labor and emotional intelligence influencing CS decision among midwives and obstetricians conducted in Kosovo, 2023 (N=155)

*Characteristics*	*Midwives (N=109)*		*Obstetricians (N=46)*	
	*n*	*%*	*n*	*%*
**Age** (years)				
≤39	62	56.88	8	17.39
40–59	39	35.78	33	71.74
60–65	8	7.34	5	10.87
**Gender**				
Male	0	0	27	58.7
Female	109	100	19	41.3
**Work experience** (years)				
≤10	72	66.06	9	19.57
11–20	25	22.94	32	69.57
>20	12	11.01	5	10.87
**Works in the private sector**				
No	95	87.16	31	67.39
Yes	14	12.84	15	32.61
**Quality of decision-making coefficient**				
Coefficient, median (IQR)	2.5	2.1–3.1	2.3	2.0–2.6
First quartile	32	29.36	14	30.43
Second quartile	17	15.6	16	34.78
Third quartile	27	24.77	11	23.91
Fourth quartile	33	30.28	5	10.87
**Emotional labour coefficient**				
Coefficient, median (IQR)	4.08	3.62–4.31	3.77	2.23–4.23
First quartile	31	28.44	11	23.91
Second quartile	24	22.02	15	32.61
Third quartile	30	27.52	13	28.26
Fourth quartile	24	22.02	7	15.22
**Emotional intelligence coefficient**				
Coefficient, median (IQR)	4.15	3.91–4.33	3.98	3.79–4.27
First quartile	26	23.85	16	34.78
Second quartile	27	24.77	12	26.09
Third quartile	26	23.85	12	26.09
Fourth quartile	30	27.52	6	13.04

IQR: interquartile range.

Among obstetricians, 19.57% had ≤10 years of experience, the majority, i.e. 69.57%, had 11–20 years, and 10.87% had >20 years. Additionally, 87.16% of midwives and 67.39% of obstetricians worked exclusively in the public sector, while 12.84% of midwives and 32.61% of obstetricians were employed in the private sector.

Table 3 shows the findings of a multilevel logistic regression comparing the clinical characteristics influencing the probability of CS decisions across maternal and obstetric characteristics that were part of clinical scenarios. Among midwives, a birth weight of 3500 g was associated with an odds ratio of 1.20 (95% CI: 1.00–1.44, p=0.054), while a birth weight of 4500 g showed an odds ratio of 1.19 (95% CI: 0.98–1.45, p=0.077). For obstetricians, these values were 1.31 (95% CI: 0.97–1.76, p=0.080) and 1.18 (95% CI: 0.93–1.49, p=0.165), respectively. A gestational length of 42 weeks was associated with a reduced likelihood of the measured outcome by midwives, with an odds ratio of 0.75 (95% CI: 0.62–0.90, p=0.002), but not by obstetricians, who had an odds ratio of 0.96 (95% CI: 0.78–1.17, p=0.669). Previous cesarean section was associated with an increased odds ratio for midwives of 1.42 (95% CI: 1.11–1.81, p=0.005) but was not significant for obstetricians, with an odds ratio of 1.31 (95% CI: 0.96–1.79, p=0.087). Hypertension was significantly associated with higher odds by midwives, with an odds ratio of 1.23 (95% CI: 1.01–1.51, p=0.042), but not by obstetricians, with an odds ratio of 1.07 (95% CI: 0.86–1.33, p=0.544). Pelvic size of 8 cm showed a stronger association in midwives, with an odds ratio of 1.70 (95% CI: 1.37–2.09, p<0.001), compared to obstetricians, with an odds ratio of 1.43 (95% CI: 0.96–2.13, p=0.078). A pelvic size of 11 cm was protective in both groups, with odds ratios of 0.73 (95% CI: 0.57–0.93, p=0.010) for midwives and 0.70 (95% CI: 0.52–0.94, p=0.019) for obstetricians. Maternal age of 40 years was significant for obstetricians, with an odds ratio of 1.43 (95% CI: 1.00–2.06, p=0.052), but not for midwives.

**Table 3:** Clinical determinants of the decision-making in a cross-sectional study of emotional labor and emotional intelligence influencing CS decision among midwives and obstetricians conducted in Kosovo, 2023 (N=155)

*Characteristics*	*Categories*	*Midwives (N=109)*			*Obstetricians (N=46)*		
		OR*	*95% CI*	*p*	OR*	*95% CI*	*p*
**Birth weight** (g)	2500 ®	1			1		
	3500	1.20	1.00–1.44	0.054	1.31	0.97–1.76	0.080
	4500	1.19	0.98–1.45	0.077	1.18	0.93–1.49	0.165
**Length** (weeks)	35	0.89	0.76–1.04	0.146	1.09	0.87–1.37	0.439
	37 ®	1			1		
	42	0.75	0.62–0.90	0.002	0.96	0.78–1.17	0.669
**Previous pregnancies**	Nullipara ®	1			1		
	Multipara	1.05	0.89–1.25	0.549	0.93	0.71–1.21	0.590
	Previous CS	1.42	1.11–1.81	0.005	1.31	0.96–1.79	0.087
**Previous condition**	None ®	1			1		
	Diabetes	1.09	0.89–1.32	0.399	1.20	0.99–1.47	0.068
	Hypertension	1.23	1.01–1.51	0.042	1.07	0.86–1.33	0.544
**Pelvic size** (cm)	8	1.70	1.37–2.09	<0.001	1.43	0.96–2.13	0.078
	10 ®	1			1		
	11	0.73	0.57–0.93	0.010	0.70	0.52–0.94	0.019
**Time of the day**	Out of office hours ®	1			1		
	Working hours	0.90	0.79–1.03	0.125	1.09	0.85–1.40	0.519
**Maternal age** (years)	18	1.06	0.88–1.29	0.531	1.29	0.97–1.74	0.085
	29 ®	1			1		
	40	1.15	0.92–1.44	0.223	1.43	1.00–2.06	0.052
**Education level**	No university degree ®	1			1		
	University degree	1.19	1.03–1.37	0.020	1.05	0.86–1.28	0.640

* Adjusted for all characteristics presented in the table.

® Reference categories.

Finally, a university education level was significantly associated with higher odds in midwives, with an odds ratio of 1.19 (95% CI: 1.03–1.37, p=0.020), but not in obstetricians, with an odds ratio of 1.05 (95% CI: 0.86–1.28, p=0.640).

Multilevel logistic regression analysis results of non-clinical determinants that influence midwives’ and obstetricians’ decisions to perform CS are presented in Table 4. Healthcare professionals aged 40–59 years, compared to those aged ≤39 years had an odds ratio of 0.95 (95% CI: 0.77–1.18, p=0.641) for midwives and 0.89 (95% CI: 0.67–1.20, p=0.450) for obstetricians. Those aged 60–65 years had an odds ratio of 0.95 (95% CI: 0.62–1.44, p=0.797) for midwives and 0.81 (95% CI: 0.50–1.30, p=0.377) for obstetricians. With respect to work experience, midwives with 11–20 years of experience, compared to those with ≤10 years, had an odds ratio of 1.11 (95% CI: 0.89–1.38, p = 0.360), as did obstetricians, with an odds ratio of 1.11 (95% CI: 0.84–1.45, p = 0.465). Those with >20 years of experience had an odds ratio of 1.05 (95% CI: 0.74–1.50, p=0.789) for midwives and 1.02 (95% CI: 0.64–1.64, p=0.923) for obstetricians. Professionals working in the private sector had higher odds of CS, with an odds ratio of 1.16 (95% CI: 0.88–1.52, p=0.286) for midwives and 1.27 (95% CI: 0.97–1.65, p=0.077) for obstetricians.

**Table 4:** Health professional’s determinants of the decision making in a cross-sectional study of emotional labor and emotional intelligence influencing CS decision-making among midwives and obstetricians conducted in Kosovo, 2023 (N=155)

*Characteristics*	*Categories*	*Midwives (N=109)*			*Obstetricians (N=46)*		
		OR*	*95% CI*	*p*	OR*	*95% CI*	*p*
**Age** (years)	≤39 ®	1			1		
	40–59	0.95	0.77–1.18	0.641	0.89	0.67–1.20	0.450
	60–65	0.95	0.62–1.44	0.797	0.81	0.50–1.30	0.377
**Work experience** (years)	≤10 ®	1			1		
	11–20	1.11	0.89–1.38	0.360	1.11	0.84–1.45	0.465
	>20	1.05	0.74–1.50	0.789	1.02	0.64–1.64	0.923
**Works in the private sector**	No ®	1			1		
	Yes	1.16	0.88–1.52	0.286	1.27	0.97–1.65	0.077
**Quality of decision-making coefficient**	First quartile ®	1			1		
	Second quartile	1.26	0.92–1.71	0.144	1.07	0.78–1.47	0.665
	Third quartile	0.94	0.73–1.20	0.611	0.98	0.74–1.29	0.876
	Fourth quartile	1.15	0.87–1.53	0.314	1.03	0.70–1.52	0.871
**Emotional labour coefficient**	First quartile ®	1			1		
	Second quartile	0.81	0.61–1.07	0.144	0.89	0.65–1.22	0.486
	Third quartile	0.85	0.65–1.11	0.235	0.86	0.61–1.20	0.380
	Fourth quartile	0.80	0.61–1.06	0.126	0.92	0.64–1.32	0.657
**Emotional intelligence coefficient**	First quartile ®	1			1		
	Second quartile	1.17	0.88–1.55	0.289	0.88	0.66–1.16	0.366
	Third quartile	1.23	0.92–1.64	0.169	1.04	0.77–1.42	0.780
	Fourth quartile	0.90	0.67–1.22	0.494	1.28	0.94–1.75	0.121

* Each characteristic is analysed as a separate multilevel random intercept model with robust variance, which includes all clinical characteristics, as shown in Table 3.

® Reference categories.

For midwives, the quality of decision-making coefficient quartiles showed no clear trend across quartiles, as the odds of CS fluctuated. The second quartile showed an increased odds ratio of 1.26 (95% CI: 0.92–1.71, p=0.144), the third quartile showed a decrease with an odds ratio of 0.94 (95% CI: 0.73–1.20, p=0.611), and the fourth quartile indicated a slight increase with an odds ratio of 1.15 (95% CI: 0.87–1.53, p=0.314). For obstetricians, we also saw no discernible trend. The second quartile showed an odds ratio of 1.07 (95% CI: 0.78–1.47, p=0.665), the third quartile decreased to 0.98 (95% CI: 0.74–1.29, p=0.876), and the fourth quartile returned to near baseline with an odds ratio of 1.03 (95% CI: 0.70–1.52, p=0.871).

In the case of midwives, the emotional labor coefficient quartiles showed a downward trend in the odds of CS as quartiles increased. The second quartile had an odds ratio of 0.81 (95% CI: 0.61–1.07, p=0.144), the third quartile was 0.85 (95% CI: 0.65–1.11, p=0.235), and the fourth quartile showed further reduction with an odds ratio of 0.80 (95% CI: 0.61–1.06, p=0.126). For obstetricians, the quartiles followed a non-linear pattern without a consistent trend. The second quartile showed an odds ratio of 0.89 (95% CI: 0.65–1.22, p=0.486), the third quartile decreased slightly to 0.86 (95% CI: 0.61–1.20, p=0.380), and the fourth quartile returned to 0.92 (95% CI: 0.64–1.32, p=0.657).

The emotional intelligence coefficient quartiles for midwives showed no consistent trend. Odds ratios increased slightly in the second quartile (OR=1.17; 95% CI: 0.88–1.55, p=0.289) and the third quartile (OR=1.23, 95% CI: 0.92–1.64, p=0.169), but then decreased in the fourth quartile (OR=0.90; 95% CI: 0.67–1.22, p=0.494). For obstetricians, a slight upward trend in odds of CS was observed as quartiles increased, with the second quartile at 0.88 (95% CI: 0.66–1.16, p=0.366), the third quartile at 1.04 (95% CI: 0.77–1.42, p=0.780), and the fourth quartile peaking at 1.28 (95% CI: 0.94–1.75, p=0.121). It is worth noting that all values were not statistically significant.

## DISCUSSION

The analysis explored the clinical and non-clinical factors influencing decisions to perform cesarean sections (CS) among midwives and obstetricians. Overall, midwives and obstetricians varied in their clinical decision-making influence. For midwives, a birth weight of 4500 g, previous CS, hypertension, and a pelvic size of 8 cm increased CS likelihood, while a gestational age of 42 weeks reduced it. Obstetricians showed fewer significant associations, with a maternal age of 40 years and a notable protective effect of larger pelvic size (11 cm). Non-clinical determinants such as age, experience, and sector showed minimal influence, with private practice slightly increasing odds for both groups. Emotional labor and emotional intelligence quartiles demonstrated inconsistent or non-significant trends for CS likelihood, though midwives showed a potential protective effect with higher emotional labor quartiles.

In light of the excess number of cesarean sections in the country, our study explored how different clinical and non-clinical patient variables and factors related to the providers shape a clinician’s decision to recommend CS. Such insight is essential to improving health service delivery in Kosovo, where healthcare reforms have attempted to enhance maternal care in the face of varying and increasing CS rates in public hospitals^6,32^. The complexity of healthcare providers’ decision-making surrounding CS has been examined in Russia by Danishevski et al.^30^. Both studies highlight the variation in practice impacted by particular medical comorbidities and clinical conditions like pelvic size. Higher birth weights are associated with increased odds of CS in both. However, the Russian study reports stronger associations (e.g. 4200 g: OR=7.39 vs about 1.19–1.31 in our study). Similarly, both studies confirm the protective effect of larger pelvic size (11 cm: OR about 0.7). Maternal age is a significant factor in both, but the Russian study shows a stronger association for older age (e.g. 32 years: OR=3.57 vs about 1.43 in our study). Our study emphasizes previous CS as a key risk factor, while the Russian study highlights specific conditions like heart disease. Our study notes the variation among scores of quality of decision-making, emotional labor and emotional intelligence coefficients. Notes consistently lower odds for CS in the case of emotional labor but are statistically insignificant. Other studies have examined the role of EI among healthcare providers, such as the 2017 study by Hutchinson et al.^33^. The authors noted that the emotional aspect of decision-making is often neglected or viewed as impeding factor in clinical decision-making processes^33^. They also reported that emotional aspects of clinical decision-making are essential skills that increased experience can strengthen^33^. Panda et al.^34^ have noted that various factors can influence clinician decisions regarding the use of CS, such as attitude toward CS, personal beliefs or perceived fear. Additionally, institutional culture can have an impact on decision-making^34^. It is worth noting that factors such as clinician gender or type can impact a clinician’s attitude towards CS^16,17^. However, individual provider characteristics are often non-modifiable, e.g. age, gender, and years of practice and understanding the interplay between these factors can help target interventions to reduce CS^35^. Education of healthcare providers and improved control of decision-making, including institutional practice patterns, and the larger role of midwives in prior and during delivery care can play a crucial role in mitigating CS overuse^11,17,36^.

### Strengths and limitations

The study’s design is the strongest point of our study. A thorough examination of existing literature and tools enabled us to identify an optimal approach for multimodal assessment of emotional labor, emotional intelligence, and decision-making, as well as conjoint analysis that examines factors influencing decisions for CS and clinician preferences. By designing and carrying out this study using the conjoint methodology, we produced attribute combinations that more effectively assess how a clinician would choose to perform a CS and which factors were more heavily weighted in these assessments. The sample was representative and included all clinicians in the public sector’s main tertiary care facility. This is among the first research initiatives to undertake a broader and context-grounded perspective of the emotional components of decision-making for the cesarean section. The application of multiple models for analyses also gave a complete and holistic examination of the interplay between emotional intelligence and emotional labor while considering multiple clinical indicators. Relying on self-reported measures of emotional intelligence and emotional labor may introduce response bias, and implicit biases related to factors such as patient education level could influence clinical decision-making in ways that surveys might not fully capture. Overall, a larger sample may have led to more valid results.

### Implications

Healthcare institutions interested in decreasing the frequency of decision-making resulting in a cesarean section might consider offering training and assistance to reduce the emotional labor of their workforce – midwives are particularly indicated in our results. Such training initiatives could also incorporate EI training for healthcare workers, although our study did not show a large impact of EI in CS decision-making. Our results show midwives and obstetricians varied in their clinical decision-making influences. Our study primarily examined factors influencing decision-making at the level of individual healthcare providers. However, we observed variation based on the sector in which obstetricians worked. In the context of Kosovo, this suggests that greater impact might be achieved through improvements in government policy and adjustments to the healthcare system.

A 2018 policy review by Harri et al.^32^ highlighted several key areas that should be addressed to improve maternal care, including CS provision. The authors highlighted the lack of standardized national CS guidelines^32^. Thus, the development and reformation of clinical protocols, as well as their implementation in professional training and health curricula, could significantly enhance maternal and neonatal health outcomes. Improved patient care and education can also lead to improved CS usage as patients become more aware of the procedure’s risks and benefits. The higher involvement of midwives in care around delivery is also known to reflect positively on the reduction of CS and higher mothers’ satisfaction^17,36^.

Our study approach considers clinical and non-clinical case parameters, as well as how emotional capabilities and individual physician characteristics influence CS. This approach, as such, gives a holistic picture of all aspects of the decision-making process at the level of the individual clinician. This gives a platform for other researchers to explore these variables further, both for CS and other clinical procedures. Adapting this design in other contexts and with larger sample sizes may provide interesting insights and add further evidence in this important domain.

## CONCLUSIONS

Numerous clinical and non-clinical factors impact the choice to have CS. Furthermore, obstetricians and midwives differ in how important these elements are when making decisions. These findings suggest that clinical decision-making for CS may not be supported by evidence. Another explanation might be that this choice is influenced by several factors outside the scope of our investigation, which calls for further investigation. These results highlight the necessity of careful consideration when developing policies and training programs that optimize cesarean section decision-making processes to improve maternal and newborn health outcomes in Kosovo and comparable contexts.

## Supplementary Material



## Data Availability

The data supporting this research are available from the authors on reasonable request.
