# Supplementary material for: The influence of emotional labor and emotional intelligence on cesarean section decision-making among midwives and obstetricians in Kosovo: A cross-sectional study using conjoint analysis
Source: Eur J Midwifery. 2025 Jan 17;9:10.18332/ejm/197168. doi: 10.18332/ejm/197168 (PMC11739932; doi:10.18332/ejm/197168)
Supplement: Supplementary file 1 [file EJM-9-05-s1.pdf]

Distribution of levels of attributes in the task matrix (orthogonal design)

Birth weight (grams)

	Freq.	Percent
2500	9	32.14
3500	9	32.14
4200	10	35.71

Length of gestation (weeks)

	Freq.	Percent
35	10	35.71
37	9	32.14
42	9	32.14

Previous Prengancies

	Freq.	Percent
Multipara	9	32.14
Nullipara	10	35.71
Previous CS	9	32.14

Existing conditions

	Freq.	Percent
None	9	32.14
Hypertension	10	35.71
Diabetes	9	32.14

Pelvic size (centimeters)

	Freq.	Percent
8	11	39.29
10	9	32.14
11	8	28.57

Time of the day

	Freq.	Percent
Out of office hours (3 pm - 7 am)	9	32.14
Working hours (7 am - 2 pm)	19	67.86

Maternal age

	Freq.	Percent
18	9	32.14
29	9	32.14
40	10	35.71

University enrolment or degree

	Freq.	Percent
No	10	35.71
Yes	18	64.29

Table with correlations among attributes for both samples (midwives and obstetricians)

Midwives

	Birth weight	Length of gestation	Previous CS	Existing conditions	Pelvic size	Time of the day	Maternal age	Level of education
Birth weight	1							
Length of gestation	-0.0517	1						
Previous CS	0	0.1082	1					
Existing conditions	0	0	0.0556	1				
Pelvic size	-0.1057	0.0414	0.0088	-0.1085	1			
Time of the day	0.0301	-0.0301	-0.0954	0	0.0101	1		
Maternal age	0.0517	-0.0517	0	0	-0.1556	0.0301	1	
Level of education	-0.0587	-0.0342	-0.093	0.093	-0.0049	0.1254	0.0342	1

Obstetricians

	Birth weight	Length of gestation	Previous CS	Existing conditions	Pelvic size	Time of the day	Maternal age	Level of education
Birth weight	1							
Length of gestation	-0.0517	1						
Previous CS	0	0.1082	1					
Existing conditions	0	0	0.0556	1				
Pelvic size	-0.1057	0.0414	0.0088	-0.1085	1			
Time of the day	0.0301	-0.0301	-0.0954	0	0.0101	1		
Maternal age	0.0517	-0.0517	0	0	-0.1556	0.0301	1	
Level of education	-0.0587	-0.0342	-0.093	0.093	-0.0049	0.1254	0.0342	1

© 2025 Taganoviq B. et al.
